# Protective effects of tea polyphenols and β-carotene against γ-radiation induced mutation and oxidative stress in *Drosophila melanogaster*

**DOI:** 10.1186/s41021-017-0084-x

**Published:** 2017-11-01

**Authors:** Isha Nagpal, Suresh K. Abraham

**Affiliations:** School of Life Sciences, Jawaharlal Nehru University, New Delhi, 110067 India

**Keywords:** *Drosophila melanogaster*, γ-radiation, Tea polyphenols, β-carotene, Sex-linked recessive lethal mutations, Antioxidant enzymes, Alternative to mammalian testing

## Abstract

**Background:**

The commonly consumed antioxidants β-carotene and tea polyphenols were used to assess their protective effects against γ-radiation induced sex-linked recessive lethal (SLRL) mutation and oxidative stress in *Drosophila melanogaster*. Third instar larvae and adult males of wild-type Oregon-K (ORK) were fed on test agents for 24 and 72 h respectively before exposure to 10Gy γ-irradiation. The treated/control flies were used to assess the induction of SLRLs. We also evaluated antioxidant properties of these phytochemicals in the third instar larvae.

**Results:**

Different stages of spermatogenesis in adult males showed a decrease in γ-radiation induced SLRL frequencies upon co-treatment with test agents. A similar trend was observed in larvae. Furthermore, a significant increase in antioxidant enzymatic activities with a decrease in malondialdehyde content was observed.

**Conclusion:**

β-carotene and tea polyphenols have exerted antigenotoxic and antioxidant effects in *Drosophila*. This study demonstrated the suitability of *Drosophila* as an alternative to mammalian testing for evaluating the antigenotoxic and antioxidant activity of natural products.

## Background

Radiations and certain chemical agents induce DNA lesions, which may cause genomic instability and activate cancer generation. Exposure to such mutagens disturbs ROS homeostasis leading to overproduction of ROS which enhances DNA damage. In the modern world scenario, exposure to such harmful radiation has become more frequent. Several studies showed that ionizing radiation (such as X-rays, γ-rays) cause lethal mutation in the germ cells of *D. melanogaster* [1–3].

Chemopreventive phytochemicals from commonly consumed fruits, vegetables and beverages are well documented for inducing antimutagenic and/or antioxidant activity by different mechanisms [4]. The most common radioprotective mode of action of phytochemicals implicate chelation and scavenging of free radicals generated during exposure to mutagens [5] and upregulation of mRNAs of antioxidant enzymes like catalase, glutathione S- transferase [6, 7] leading to overexpression of such antioxidant enzymes in vivo*,* and rescuing cells from oxidative stress and DNA damage [8].

β-carotene (BC) belongs to the carotenoid family with potential to scavenge singlet oxygen species/free radicals [9]. It is a natural precursor of vitamin A, which is most efficiently converted into vitamin A as compared to other provitamins [10]. BC is a potent antioxidant, and studies demonstrate that BC significantly reduces radiation-induced DNA damage including γ-radiation [11] and UV radiations [12]. BC is reported to protect against chromosomal damage, induction of micronuclei [13] and lipid peroxidation [14, 15].

Tea is the most common beverage consumed as green tea or black tea. Both forms of tea contain active ingredients commonly called as tea polyphenols which are strong antioxidants and exert antimutagenic potentials [16–19]. Radioprotective effects of tea polyphenols against harmful effects of radiations are widely reported in various in vitro and in vivo studies [20–24]. Studies have demonstrated that epigallocatechin gallate (EGCG), a bioactive phytochemical present in green tea can reduce UV radiation induced DNA damage in cultured human cells and peripheral leucocytes [25].

In toxicological research, animal models are widely used. However, its cost and licensing law issues limit its use in large-scale drug screening process [26]. To minimize the use of higher animals, use of alternative models have gained much importance. *Drosophila*, an invertebrate model with its extensively studied genome, shows more than 70% of gene homology with humans [27, 28]. The similarity of metabolic pathways between *Drosophila* and mammals has encouraged the use of *Drosophila* in the context of screening and evaluating the antimutagenicity especially for detecting somatic and germ cell mutations of pure and crude mixtures of various compounds [29–35]. This model has fewer ethical concerns due to which it is included in the recommendations of European Center for Validation of Alternative Methods (ECVAM) and Organization for Economic Cooperation and Development (OECD) for genotoxicity testing that promotes to reduce, refine or replace (3R’s) the use of vertebrate models for laboratory experiments [36, 37].

In *Drosophila*, genotoxic damage can be detected either by performing the somatic mutation and recombination test (SMART) or the sex-linked recessive lethal test (SLRL). The sex-linked recessive lethal (SLRL) test in *Drosophila melanogaster* is widely used for detection of genetic lesions in germ cells [38]. It can be used to study the genotoxic effects of various environmental toxicants [39–41]. This test can be used to screen recessive lethal mutations at almost 800 different loci on the X chromosome, which represents 80% of the X chromosome and one-fifth of the entire *Drosophila melanogaster* genome [42]. Since *Drosophila* model exhibits the significant activity of xenobiotic drug metabolizing enzymes like cytochrome P-450 and aryl hydrocarbon hydroxylase [43, 44], the SLRL test can be used to detect mutagens and promutagens with very short half-lives [45–47]. Promutagens are mostly activated in spermatids [48], and SLRL can be used to analyze induced mutations in specific stages of germ cells [49].

Considering the well-established antioxidant properties of BC and TP in other in vivo models, the present investigation was initiated with the main objective of assessing whether or not these test agents show a similar level of antimutagenic and antioxidant potential in the *Drosophila* model. In accordance with these objectives, the experiments were carried out to study: (a) antigenotoxic potentials of BC and TP against γ-radiation-induced SLRL mutation in the germ cells of both larvae and adult flies of *D. melanogaster,* (b) analysis of antioxidant capacity (in terms of antioxidant level/activity) following induction of oxidative stress and its modulation with phytochemicals.

## Methods

### *Drosophila* stocks

The experiments were carried out using Muller-5 or Basc stock *In(1)s* ^*s1L*^ *sc* ^8*R*^ + *S*, *sc* ^*s*1^ *sc* ^8^ *w* ^*a*^ *B*) and Oregon-K (wild type). These stocks were obtained from *Drosophila* stock center, Department of Zoology, Mysore University (India). Flies were cultured on the standard *Drosophila* culture medium [2].

### Chemicals

The chemicals used in the present studies were polyphenon 60 from green tea (CAS No.138988–88-2), and β-carotene (CAS No.7235–40-7) obtained from Sigma-Aldrich Company, India. All the other used reagents and chemicals were of analytical grade, procured from local sources.

### Irradiation

Culture vials containing adult flies and larvae of *Drosophila* were exposed to 10Gy γ-radiations at a dose rate of 1.8Gy/min in a gamma chamber (source ^60^Co, 204 TBq, 5500 Ci) obtained from Bhaba Atomic Research Centre (BARC), Mumbai, India. The dose rate was determined using Fe^2+/^Fe^3+^ dosimetry.

### Test concentrations and treatment

*Drosophila* larvae and adult flies were fed on different concentrations of test agents. The concentrations which did not show lethality or delay in development of larvae were selected for the experiments.

### Test for detecting sex-linked recessive lethal mutation

#### Adult feeding experiments

Four days old adult male flies (ORK) were starved for 6-8 h and transferred into a glass vial containing filter paper soaked in the test agents mixed with 10% sucrose solution which was also used as a negative control. The filter paper was renewed once every 12 h. After feeding on test solutions for 72 h, the control/ treated flies were irradiated, and each irradiated male was allowed to mate with 5 virgins *Basc* females for three days resulting in brood I. After three days, the same male fly was allowed to mate with another set of 5 virgin females (Muller-5) to produce brood II. The same pattern was repeated until brood IV to check the antimutagenic effect of test agents in postmeiotic (spermatozoa and spermatids), meiotic (spermatocytes) and premeiotic (spermatogonia) cells against the group treated with γ-irradiation alone [50].

#### Larval feeding experiments

Inseminated females (ORK) were allowed to lay eggs on the culture medium for 8-10 h. After this, the females were discarded, and 3 days later, the larvae were transferred to glass vials containing test solution mixed with instant *Drosophila* culture medium. After 24 h, one batch of third instar larvae was harvested and irradiated to check the enzymatic activity. The emerging one-day old adult males were mated individually with a set of 3 to 5 Basc females in order to conduct SLRL test [47].

For both the adult and larval feeding experiments, the inseminated females (one female/vial) from the F_1_ generation were used to raise the F_2_ generation which was scored for the absence of wild-type males indicating the occurrence of SLRL mutation [51].

#### Assays for oxidative stress markers

Biochemical assays were carried out for evaluating oxidative stress in control and treated third instar larva. For this purpose, larval tissue homogenates were prepared following the method by Singh et al. [52]. Protein content in cytosolic and microsomal samples was estimated by the method of Lowry et al. [53] using Folin’s reagent and BSA as the external standard. Protein concentration was expressed as mg/ml homogenate. GSH content was measured by the method of Ellman et al. [54] with some minor modifications. The reaction was monitored at 412 nm, and the GSH content was expressed as nmol/mg larval protein. Catalase activity was estimated as described by Sinha [55] and it is defined as the ability to dissociate H_2_O_2_ in 1 min of incubation time. Enzyme activity was expressed as μmol H_2_O_2_ decomposed/min/mg/larval protein. Glutathione S-transferase (GST) activity was measured by the method of Habig et al. [56] with some minor modifications using 5 mM CDNB as substrate. The formation of CDNB-GSH conjugate was measured by monitoring the absorbance for 3 min at 340 nm in a time interval of 3 s. GST activity was expressed as nmol CDNB reduced/min/mg protein using molar extinction coefficient of 6.25 × 10^3^ M^−1^ cm^−1^. Superoxide dismutase (SOD) activity was determined as described by Nishikimi et al. [57] with minor modifications as described by Singh et al. [52]. One unit of enzymatic activity is defined as the protein concentration required for inhibiting the chromogen production by half in a time interval of 1 min, and the SOD activity is expressed as U/min/mg protein. Malondialdehyde (MDA) content was measured as a marker of lipid peroxidation (LPO) by the method of Okhawa et al. [58]. MDA was assayed using tetra ethoxy propane as an external standard. It was expressed in nmoles MDA formed/h/mg protein.

The data is presented as percentage change by using the formula:

$$ \mathrm{Mean}\ \left(\mathrm{sample}\right)-\mathrm{Mean}\ \left(\mathrm{Control}\right)/\mathrm{Mean}\ \left(\mathrm{Control}\right)\ \mathrm{x}\ 100 $$

### Statistical analysis

The experimental values are represented as mean ± SD. Statistical analysis was carried out using one-way analysis of variance (ANOVA) followed by post hoc tests for multiple comparisons among the treated groups and control using Newman’s Keuls test. Pearson’s correlations were also calculated and analyzed using Prism computer program (GraphPad version 5.0, San Diego, CA, USA). Kastenbaum and Bowman tables were used for validation of results obtained from SLRL test [59, 60], which are further analyzed by the Z Test for difference in proportions.

## Results

### Induction of SLRLs by 10Gy γ*-*radiation in *Drosophila* larvae and adult flies and its modulation by BC and TP

In our study, we examined the antimutagenic potential of BC and TP against γ-radiation-induced SLRL mutation. As compared to negative control group, no significant difference was observed in the mutation frequencies of individual treatment of TP and BC group. However, the mutation frequencies significantly increased with the positive control group (10Gy γ–radiation).

We observed a significant reduction in the incidence of mutation frequencies with phytochemical pre-treatment against γ-radiation induced mutation in the germ cells of larvae as well as adult flies of *D. melanogaster.* The results obtained in terms of % lethals from the adult and larval SLRL experiments with the two test compounds are summarized in Tables 1 and 2 respectively.

**Table 1:** Adult feeding experiments: Induction of sex-linked recessive lethals (SLRLs) by 10Gy γ-radiation and its modulation by TP and BC in *Drosophila* adult males

Treatment	Brood	No: of X-Chromosomes scored	Lethals (n)	Lethals (%)	
10% Sucrose (Negative Control)	I	913	1	0.110	
	II	837	2	0.239	
	III	682	1	0.147	
	IV	554	1	0.181	
	**I-IV**	**2986**	**5**	**0.167**	
TP 1%	I	781	2	0.256	
	II	746	1	0.134	
	III	614	1	0.163	
	IV	517	0	0.000	
	**I-IV**	**2658**	**4**	**0.150**	
BC 0.25%	I	869	1	0.115	
	II	684	2	0.292	
	III	577	0	0.000	
	IV	461	1	0.217	
	**I-IV**	**2591**	**4**	**0.154**	
BC 0.5%	I	889	2	0.225	
	II	702	1	0.142	
	III	693	0	0.000	
	IV	415	1	0.241	
	**I-IV**	**2699**	**4**	**0.148**	
BC 1%	I	902	2	0.222	
	II	819	1	0.122	
	III	674	1	0.148	
	IV	529	1	0.189	
	**I-IV**	**2924**	**5**	**0.171**	
γ-radiation	I	752	31	4.122^###^	
	II	732	27	3.689^###^	
	III	717	15	2.092^###^	
	IV	665	12	1.805^###^	
	**I-IV**	**2866**	**85**	**2.966** ^###^	
γ-radiation + TP 1%	I	825	8	0.970^***, ##^	{76.46%}
	II	782	5	0.639***	{82.67%}
	III	645	5	0.775^**, #^	{62.95%}
	IV	562	4	0.712^*^	{60.55%}
	**I-IV**	**2814**	**22**	**0.782** ^***, ###^	**{73.63**%}
γ-radiation + BC 0.25%	I	810	7	0.864^***, ##^	{79.03%}
	II	711	6	0.844^***, #^	{77.12%}
	III	512	4	0.781^*, #^	{62.66%}
	IV	499	3	0.601^*^	{66.70%}
	**I-IV**	**2532**	**20**	**0.790** ^***, ###^	**{73.36**%}
γ-radiation + BC 0.5%	I	788	7	0.888^***, ##^	{78.45%}
	II	651	5	0.768^***^	{79.18%}
	III	582	4	0.687^**^	{67.16%}
	IV	558	4	0.717^*^	{60.27%}
	**I-IV**	**2579**	**20**	**0.775** ^***, ###^	**{73.87**%}
γ-radiation + BC 1%	I	704	8	1.136^***, ###^	{72.44%}
	II	688	7	1.017^***, ##^	{95.36%}
	III	662	7	1.057^**##**^	{49.47%}
	IV	522	4	0.766	{57.56%}
	**I-IV**	**2576**	**26**	**1.009** ^***, ###^	**{65.98**%}

* p ≤ 0.10, ** p ≤ 0.05, *** p ≤ 0.01 (Compared to positive control group. i.e. γ-radiation)

*#p ≤ 0.10, ## p ≤ 0.05, ###p ≤ 0.01* (Compared to negative control group. i.e. sucrose control)

The data in bold indicates the sum of all four broods for different test groups respectively

The percentages in parentheses indicates the decrease rates in the mutation frequency of test groups as compared to positive control group

**Table 2:** Larval feeding experiments: Induction of sex-linked recessive lethals (SLRLs) by 10Gy γ- radiation and its modulation by TP and BC in *Drosophila* larvae

Treatment	No: of X- chromosomes scored	Lethals (n)	Lethals (%)	
Control	1808	4	0.221	
TP 1%	1095	2	0.183	
BC 0.25%	935	2	0.214	
BC 0.5%	1167	3	0.257	
BC 1%	1218	4	0.328	
γ-radiation	1315	27	2.053^###^	
γ-radiation + TP 1%	1121	13	1.160^***, ###^	{43.49%}
γ-radiation + BC 0.25%	986	12	1.217^***, ###^	{40.72%}
γ-radiation + BC 0.5%	885	11	1.243^***, ###^	{39.45%}
γ-radiation + BC 1%	722	10	1.385^***,###^	{32.53%}

* p ≤ 0.10, ** p ≤ 0.05, *** p ≤ 0.01 (Compared to positive control group. i.e. γ-radiation)

*#p ≤ 0.10, ##p ≤ 0.05, ###p ≤ 0.01* (Compared to negative control group. i.e. sucrose control)

The percentages in parentheses indicates the decrease rates in the mutation frequency of test groups as compared to positive control group

Data from adult feeding experiments (Table 1) demonstrates that BC (0.5%) + γ-radiation has significantly reduced the mutation frequency induced by 10 Gy γ-irradiation in all the germ cell stages (Brood I-IV) from 2.966 to 0.775% (73.85% reduction, *p ≤ 0.01*) followed by γ-radiation + TP (1%) which is 2.966 to 0.782% (73.64% reduction, *p ≤ 0.01*). Results obtained from larval feeding experiments (Table 2) showed γ-radiation + TP (1%) has significantly reduced the mutation from 2.053 to 1.160% (43.51% reduction, *p ≤ 0.01*) followed by γ-radiation + BC (0.25%), in which the reduction was from 2.053 to 1.217% (40.72% reduction, *p ≤ 0.01*).

### Modulation of oxidative stress levels in third instar larvae of *D. melanogaster*

As compared to negative control group, the amounts of anti-oxidant enzymes (GSH, GST, CAT) slightly but significantly increased and the LPO level decreased when treated with TP 1%, and GSH increased with BC 0.25%. However, the amounts of anti-oxidant enzymes significantly decreased and the LPO level increased when treated with γ-radiation (10Gy).

As compared to positive control group (10 Gy γ-irradiation), co-treatment of test agents with γ-radiation has shown a significant induction in antioxidant enzymatic activities. When the larvae were exposed to γ-radiation + TP (1%), SOD activity has increased up to, 100.37% *(p ≤ 0.001*) > CAT (71.94%; *p ≤* 0.001) > GST (52.86%; *p ≤* 0.001). Co-treatment with γ-radiation + BC (0.25%) has increased the activities of SOD and CAT by 102.09% and 65.18% respectively *(p ≤ 0.*001) with maximum GST activity (53.53%; *p ≤* 0.001) when the larvae were treated with γ-radiation + BC (0.5%) (Table 3).

**Table 3:** Modulatory effects of TP and BC against γ-radiation induced oxidative stress on third instar larvae of *D. melanogaster* (ORK)

Parameters	^a^GSH	^b^GST	^c^CAT	^d^SOD	^e^LPO
Control	62.69 ± 3.66	37.39 ± 4.75	54.56 ± 4.30	3.56 ± 0.17	2.48 ± 0.30
TP 1%	76.56 ± 2.73^###^	46.28 ± 2.29^#^	64.68 ± 2.89^#^	3.95 ± 0.25	1.74 ± 0.21^#^
BC 0.25%	71.87 ± 1.58^##^	43.67 ± 3.72	53.01 ± 2.65	4.07 ± 0.10	2.07 ± 0.20
BC 0.5%	64.32 ± 3.24	41.12 ± 2.08	57.53 ± 4.50	3.74 ± 0.11	2.24 ± 0.22
BC 1%	64.26 ± 1.67	35.99 ± 4.79	51.91 ± 3.14	3.59 ± 0.21	2.08 ± 0.23
γ-radiation	49.41 ± 1.89^###^	23.63 ± 2.94^###^	31.08 ± 5.90^###^	1.45 ± 0.34^###^	5.70 ± 0.30^###^
γ-radiation + TP (1%)	61.97 ± 2.31^***^ {25.41%}	36.12 ± 2.55^**^ {35.63%}	53.44 ± 4.08^***^ {71.94%}	2.89 ± 0.22^***, ##^ {99.31%}	3.94 ± 0.17^***, ###^ {- 30.87%}
γ-radiation + BC (0.25%)	58.07 ± 1.80^**^ {17.52%}	34.28 ± 1.63^**^ {28.72%}	51.34 ± 2.56^***^ {65.18%}	2.93 ± 0.26^***, ##^ {102.0%}	2.48 ± 0.29^***^ {- 56.49%}
γ-radiation + BC (0.5%)	57.51 ± 2.18^**, #^ {16.39%}	36.28 ± 4.03^**^ {28.72%}	50.71 ± 4.28^***^ {63.15%}	2.51 ± 0.27^***, ###^ {73.10%}	2.60 ± 0.23^***^ {- 54.3%}
γ-radiation + BC (1%)	52.37 ± 2.67^***, ###^ {5.99%}	29.67 ± 3.08^*^ {11.41%}	38.52 ± 4.42^*, ##^ {23.93%}	2.20 ± 0.24^***, ###^ {51.72%}	2.84 ± 0.21^***^ {- 50.17%}

Effect of TP and BC on (^a^) Reduced glutathione (GSH) levels expressed as nM of GSH formed mg^−1^ protein. (^b^) Glutathione S-transferase (GST) activity expressed as nM of CDNB conjugated min^−1^ mg^−1^ protein. (^c^) Catalase (CAT) activity expressed as μmoles of H_2_O_2_ decomposed min^−1^ mg^−1^ protein. (^d^) Superoxide dismutase (SOD) activity expressed as units min^−1^ mg^−1^ protein. (E) Lipid peroxidation (LPO) expressed as nmol of MDA formed h^−1^ mg^−1^ protein. Data represented as mean ± SD of three identical experiments. ^***#***^ *p ≤* 0.05; ^***##***^ *p ≤* 0.01; ^***###***^ *p ≤* 0.001 compared with normal control group; *p ≤ 0.05; **p ≤ 0.01; ***p ≤ 0.001 compared with γ-radiation treated group

The percentages in parentheses indicates the increase rates in the GSH, GST, CAT and SOD activity and decrease (-) in the LPO of test groups as compared to positive control group

### Modulatory effects of BC and TP on GSH and LPO levels in *Drosophila* larvae

A significant increase in glutathione levels (GSH) and reduction in MDA content (a measure of lipid peroxidation) was observed in the treated larvae as compared to positive control group (10Gy γ-radiation). Maximum GSH content was observed in the group where larvae were exposed to γ-radiation + TP (1%) (25.42% increase) followed by γ-radiation + BC (0.25%) (17.52% increase). γ-radiation + BC (0.25%) has reduced the MDA content by 56.5% followed by 54.4% reduction in γ-radiation + BC (0.5%) (Table 3).

### Correlation between different stress parameters

A correlation was drawn between the different sets of oxidative stress markers (GSH, GST, CAT, SOD, and LPO) when the larvae were exposed to test agents (TP and BC) individually as well as in combination with γ-radiation. A significant negative correlation of MDA content (lipid peroxidation) and positive correlation of GSH content was observed with different sets of antioxidant enzymes (GST, CAT, and SOD) (Tables 4 and 5).

**Table 4:** Correlation between oxidative stress parameters. Correlation between different sets of oxidative stress markers (GSH, GST, SOD, CAT and LPO) when the larvae were exposed to TP and combination of TP (1%) + γ-radiation (10Gy)

	GSH	GST	CAT	SOD	LPO
GSH	1				
GST	0.989	1			
CAT	0.959	0.990	1		
SOD	0.925	0.966	0.975	1	
LPO	−0.927	−0.955	−0.948	−0.990	1

**Table 5:** Correlation between different sets of oxidative stress markers (GSH, GST, SOD, CAT and LPO) when the larvae were exposed to varying doses of BC and combination of BC (0.25%, 0.5%, 1%) + γ-radiation (10Gy)

	GSH	GST	CAT	SOD	LPO
GSH	1				
GST	0.938	1			
CAT	0.820	0.917	1		
SOD	0.965	0.934	0.896	1	
LPO	−0.743	−0.842	−0.863	−0.826	1

## Discussion

Ionizing radiations are deleterious in nature and cause direct and indirect DNA damage. In recent years, human exposure to harmful levels of radiations has increased due to radiation based clinical diagnosis, and radiotherapy for cancer treatment [61]. Also, studies have shown that radiotherapy is responsible for “bystander effect” [62]. Ionizing radiation starts a cascade of events which leads to free radical generation [63]. DNA damage can occur in many different ways, amongst which adduction of free radicals with DNA is most common under stressful conditions [64]. Hence, to prevent free radical associated DNA damage, the most common strategy is to quench the generated free radicals. In the present study, we observed the antimutagenic and antioxidant activity of TP and BC against γ-radiation induced mutation and oxidative stress in the larvae and adult flies of *D.melanogaster*.

For testing γ-radiation induced mutation in the germ cells, SLRL test was performed to assess radioprotective effects of TP and BC in both larvae as well as adult flies of *D. melanogaster*. The maximum reduction of mutation frequencies in larvae was observed with γ-radiation + TP (1%), followed by γ-radiation + BC(0.25%) when compared to 10Gy γ-radiation. In the case of adult germ cells, a significant reduction was observed in all the successive germ cell stages (i.e. Brood I to IV) when flies were treated with γ-radiation + BC (0.5%) followed by γ-radiation + TP (1%). TP and BC have shown antigenotoxic activity against various chemical carcinogens in *Drosophila* [65, 66]*.* Significant protective effects of TP and its active constituents have been demonstrated against γ-radiation induced DNA damage in human lymphocytes [67] and also in splenocytes [68] and blood leucocytes [68] of mice. Similarly, BC showed protective effects against damage induced by X-rays and γ-rays in germ cells and somatic cells of rats and mice [11, 13, 69].

Radiation induced reactive oxygen species (ROS) is a major cause of oxidative stress to cells. Oxidative stress arises due to the imbalance of free radical generation and the antioxidant defense mechanisms. TP and BC are potent scavengers of free radicals. Our result show that pre-treatment with TP and BC has inhibited the reduction in antioxidant enzyme levels (GSH, GST, SOD, and CAT) caused by exposure to γ-radiation. Furthermore, this pre-treatment has rescued the cells from damage caused by lipid peroxidation. These findings are in agreement with in vivo and in vitro studies in which pre-treament with TP and its active constituents led to a reduction in LPO levels [70] with a concomitant increase in intracellular levels of GSH, GST, CAT and SOD [68, 71–75]. A similar trend was observed for BC in humans [76, 77] and experimental animals [11].

Administration of these two phytochemicals (TP and BC) has reduced γ-radiation induced oxidative stress up to >50%. Furthermore, the antioxidant activity results are correlating well with the SLRL data we obtained. Since TP and BC are strong antioxidants, the most plausible explanation for the observed antimutagenic and antioxidant activity against γ-radiation could be the free radical scavenging property of these dietary phytochemicals. The antioxidant activities of TP and BC are attributed to their chemical nature. Chemically, TP contains phenolic structure that promotes electron sharing with free radical and exhibits the process of electron resonance dissociation [78], while BC contains conjugated alkyl structure enabling it to trap and stabilize peroxy free radicals and thereby reduce damage to the cell and cell membrane [79].

An important observation from the present study is the absence of a dose response for the antigenotoxic and antioxidant activity of BC. The highest dose of BC (1%) showed a lower level of antimutagenicity and antioxidant activity as compared to the other two doses (BC 0.25%) and (BC 0.5%). These observations suggest the possibility of a biphasic effect of BC that showed protection when taken at dietary levels but may have adverse effects when consumed in higher doses [80–82].

The present work further highlights the utility of *Drosophila* for evaluating the genotoxicity and antigenotoxicity of single as well as crude mixtures of dietary phytochemicals. The suitability of *Drosophila* for detecting antigenotoxic effects of coffee, a complex mixture of bioactive compounds, was first demonstrated in our laboratory [83, 84] using the somatic mutation and recombination test (SMART). Subsequently, many publications have shown the suitability of *Drosophila* for detecting antigenotoxic effects of crude mixtures of natural compounds [85–87]. Our studies were further strengthened by the similarity between the results of *Drosophila* and other in vivo and in vitro systems on antimutagenic effect and antioxidant activity of TP and BC. To the best of our knowledge, so far no study has been performed using SLRL test to assess the protective efficacy of BC and TP against γ-radiation-induced mutation and oxidative stress in the germ cells of larvae and adult flies of *D*.*melanogaster*.

## Conclusion

In conclusion, our present work has demonstrated the important role *Drosophila* assays can play in assessing the antimutagenic and antioxidant activity of pure compounds and crude mixtures of dietary phytochemicals in different germ cell stages. The similarity and correlation of experimental data between *Drosophila* and in vivo mammalian assays further reveal the usefulness of this test system for extrapolation to mammals indicating that the *Drosophila* test system is a favourable candidate to be considered as an alternative to mammalian testing.

## Abbreviations

BC: β carotene
CAT: Catalase
GSH: Glutathione content
GST: Glutathione S-transferase
LPO: Lipid peroxidation
ROS: Reactive oxygen species
SOD: Superoxide dismutase
TP: Tea Polyphenon-60
